# Back to Work

**Published:** 1947

**Authors:** H. Chitty

**Affiliations:** Hon. Consulting Surgeon, Bristol Royal Hospital


### The Bristol Medico-Chirurgical Journal

*"Scire est nescire, nisi id me*
*Scire alius sciret."*

SPRING, 1947.

### BACK TO WORK

The Presidential Address, delivered on Wednesday, 9th October, 1946, at the opening of the Sixty-eighth Session of the Bristol Medico-Chirurgical Society.

BY

MR. H. CHITTY, M.S., F.R.C.S.

*Hon. Consulting Surgeon, Bristol Royal Hospital.*

The subject of this address has been suggested by the difficulties accompanying our present change-over from war to peace, accentuated as these are by our being a debtor instead of a creditor nation. No longer have we the accumulated savings of our forebears to fall back upon in times of stress ; henceforth our standard of living must depend entirely upon our own efforts.

The late world conflict has left behind it a spirit of violence and suspicion, while we ourselves are surrounded by those whose homes and lives have been wrecked by warfare. One thinks of earlier days as having been stable and free from such menaces, yet for many those days were even less secure than are the present. Poverty and unemployment were rife throughout the industrial age, and I believe that it was mainly fear of a return to pre-war conditions which led to the great swing to the left in the recent parliamentary elections.

I want to consider how we, as medical men and women, can help to keep our people regularly at work. There are, of course, many factors, national and international, over which we have no control ; but in the realms of education and industry we can exert a great influence.

Turning first to education : It seems to me that upon this rests our greatest hope, for by increased efficiency alone can we hold our own in world trade, while the requisite skill can only be obtained by better general and technical education. It grieves me to see children at the age of fourteen taken from school and put into some blind-alley occupation in order that they may add a few shillings to the family budget. If one proposed landing a commando on an enemy coast, one would not take the first hundred soldiers available and push them forthwith into a vessel for transport to the other side. Every man would be carefully selected for his mental, moral and physical suitability and would then be submitted to intensive training. Our children need the same preparation for the battle of life.

I want to see much more use made of the medical psychologist, so that every child may be helped to find the class of work for which he is best suited, and then receive proper training for that work. Such training and subsequent placement should be available for *all* children, including the physically handicapped ; for the pioneering work of Dame Agnes Hunt, Sir Robert Jones and others, has shown how few there are, whatever their initial disablement, who cannot become self-supporting. Many a properly trained cripple leads a fuller life and earns a much better income than does the untrained general labourer, however physically fit. An effort to secure better education I regard as our first aim.

There cannot be many people, however, who get through life without being thrown out of work—at least temporarily—by some sickness or injury. In the more serious cases it depends largely upon us medical men how soon the patient gets back to work, or indeed whether he gets back to work at all; so it will be worth while surveying some modern developments which help his speedy recovery. The contrast between the results obtained in the treatment of the wounded in the first and second world wars was almost unbelievable. This was chiefly due to the control of sepsis, but improved fracture technique and the great development which had taken place meanwhile in "rehabilitation" methods, mainly in connection with our fracture clinics, were also largely responsible.

Until the first war, it seems to me, the treatment of a fracture differed little from that of mediaeval times. In our hospitals I am afraid that most surgeons regarded a fracture as an uninteresting case which blocked a bed : so a newly qualified House Surgeon treated it more or less by the light of Nature, and shot it off home or to a poor-law institution as soon as it was fit to be moved. There were few physiotherapy departments attached to our hospitals, and in them treatment consisted of little beyond massage. Only slowly did the Scandinavian "Swedish exercises" spread to this country, but it is upon their underlying principles of active movement and active co-operation upon the part of the patient in his own treatment that most of our modern advances depend.

The 1914–18 war saw the birth of occupational therapy, which depends for its success upon the more or less unconscious use of muscles and limbs. It is quite true that a man may be able to develop his calf muscles (for instance) by rising repeatedly upon tip-toe, but after five or ten minutes of this he is bored stiff; if, however, he performs the same movement when working a treadle which turns a lathe or works a fretwork machine, his mind is concentrated upon the object which he is creating, and he will as likely as not carry on for hours on end.

Occupational therapy has become a fine art and some of its exponents are most ingenious in altering and adapting the same machine to exercise different groups of muscles and even different limbs, while gradually extending the range of joint movements. I am glad to say that the Ministry of Health is encouraging the provision of this class of work in all our large hospitals. For those confined to bed for any length of time the teaching of simple handicrafts undoubtedly helps to keep up the spirits and introduces into the wards a cheerful atmosphere which is extremely beneficial. In the case of ambulant patients, organized classes and games make a change from work and provide healthy competition and companionship.

Warfare, during which large bodies of men are assembled in a single service, whose lives are under the complete control of one command, does encourage the initiation and development of experiments on a grand scale. From the three services we have of late years learned a lot about the restoration to health of the injured and the unfit. Many excellent papers have appeared in our journals. I cannot do better than quote the figures given by Air Commodore Clarke[1] of the results obtained by the R.A.F. Orthopædic Rehabilitaton Service. Of those injured over a period of five years, and this of course includes many bad smashes and multiple injuries, 77 per cent. returned to full duty; 18 per cent. returned to modified duty or were retrained; while less than 5 per cent.. were invalided out as being unfitted physically or psychologically for the service.

In civilian life the great shortage of man-power has led to similar developments and equally gratifying results. The vast majority of people who have been brought back to a fairly normal state after a period of incapacity return to their old jobs or manage to find similar ones through the labour exchanges. Others will be able to resume their former occupation in time, but need to go easy at first. We have all of us signed up such people as "fit for light work only." Too often such light work has not been available and we must have constantly noted the physical, mental and moral degeneration which ensues—the disastrous effect of prolonged idleness.

Interesting work is being done in this country and in others to get such handicapped persons back into employment. In Russia all big works are required by law to provide special departments to assist in the absorption of these cases. Under medical supervision men and women are given jobs to do for a prescribed number of hours: where required, machines may be run at half-speed or otherwise modified. In this way the works themselves provide occupational therapy. Similar methods are adopted in this country by the Austin works in association with the Birmingham Accident Hospital. The Miners' Welfare Association and the Manchester Docks have analogous schemes for getting men reabsorbed. I feel that there is room for extension of such plans to many other industries so that there shall be no gap between the end of medical treatment and the recommencement of work. We *must* try to get this idea home to our industrialists.

Next we come to those who are permanently disabled from resuming their former occupations. As you know, such people are encouraged to enrol upon the register of the disabled and all works employing upwards of twenty men must engage 3 per cent. of their employees from persons upon this register. This may not sound too easy, but a careful analysis shows what a large number of things can be done perfectly well by persons having some physical disablement. At the Ford works a survey showed that many jobs could be carried out just as well by cripples, amputees and even the blind as by those in the A1 class. As a result, about 10 per cent. of those employed in these works are physically defective, included among them epileptics and tuberculous persons. Mr. Ford himself says, "no one applying for work is refused on account of his physical condition," and "there are more places that can be filled by cripples than there are cripples."[2] A similar analysis is being carried out by some firms in this country and certain jobs are being reserved for the disabled.[3] In the U.S.A. cards have been issued on which can be marked the physical demands of each job, and similar cards upon which can be entered the capabilities of the disabled person. Thus one can almost automatically fit the man and the job.[4]

In this country, when any service patient is admitted to an E.M.S. or civilian hospital, he is put in contact by the Medical Officer in charge of the case with an official of the Ministry of Labour known as the Disablement Rehabilitation Officer, who interviews the man and finds out all about his former occupation and training. On the patient's discharge from hospital it is the duty of this official to put him into touch with suitable work. To assist in this task a vocational guide is being compiled indicating the exact requirements of various trades. The D.R.O. has the advantage of advice from the Hospital M.O. and from the patient's private doctor, who is given a summary of the hospital notes. This service is also available for civilian cases, and more use should be made of it than is the case at present. It is to be hoped that ere long such officials will have to submit to as complete a training as do our Hospital Almoners, whose work is in many respects similar.

It may be found that a man would be suitable for certain forms of employment provided that he had the necessary knowledge and skill. For such cases the Government has set up a number of training centres where upwards of forty different trades may be learned, the usual duration of the course of instruction being six months. The number of centres and the number of trades is being increased, but there seems to be a long waiting-list at the moment. There are three residential training centres for those badly disabled persons who require training in special trades. Here again there is room for a great expansion. At present it is difficult to secure admission and a long and discouraging wait results.

There are, of course, some who are so severely disabled that they can never hope to compete in the open market. A good many sufferers from medical disabilities come under this heading, e.g., cardiac cases, some asthmatics, and others who suffer from chronic and recurrent chest conditions; also epileptics and the mentally subnormal. Many of these can do work, as has been proved by the excellent results obtained with the tuberculous in the Papworth Colony. But they do need sheltered conditions. For them very little has been done in the past, and this entirely by voluntary agencies, e.g., the Lord Roberts Memorial Workshops. But provision is to be made under the Disabled Persons (Employment) Act for the setting up of non-profit-making companies to secure their training and employment, and government grants may be made to bodies already in existence for their upkeep and extension.

To study the whole problem and to extend help to such cases the "Disabled Persons Employment Corporation" was formed last year under government auspices. It has very wide powers and can provide workshops, hostel accommodation and special facilities for home workers. I understand that three factories have already been set up by this body and are in active production, and that more are to be established shortly. All the methods of securing employment which I have mentioned are available to women exactly as they are to men.

As you see, many developments are taking place and all of us should keep abreast with them and make full use of the facilities which do already exist. In this way we shall benefit the whole nation and we shall prevent much unhappiness by hastening the day when our individual patients get "Back to Work."

REFERENCES

1 *Lancet*, May 18th, 1946, p. 721.

2 *My Life and Work*, by Henry Ford.

3 Disabled Persons (Employment) Act, 1944.

4 I.L.O., *Training and Employment of Disabled Persons*, Appendix X, p. 293.

